# Supplementary material for: Using refined methods to pick up mice: A survey benchmarking prevalence & beliefs about tunnel and cup handling
Source: PLoS One. 2023 Sep 7;18(9):e0288010. doi: 10.1371/journal.pone.0288010 (PMC10484434; doi:10.1371/journal.pone.0288010)
Supplement: Attachment — Submitted filename: Comments to the author.pdf [file pone.0288010.s003.pdf]

This submission represents a survey of current mouse handling practices, primarily in the USA, and concludes that much additional education is needed to promote wider use of refined handling. While I agree with the methods and conclusions, my only major grip is that statement to that effects are repetitive, especially in the discussion and what is said in the final conclusion. I feel the manuscript could be significantly shorter and the wording more concise. Other issues to address are listed below, but these are generally only minor issues.

The figures in Table 1 under role add to 98% not 100%. Are these rounded, if not, what happened to the missing 2%?

In the individual practices section, it is stated that 215 participants reported on the methods they personally use to pick up mice. Why then, does Figure 5 only report data from 194 participants? Was this just because not all answered those questions?

Lines 423-424 say participants that answered the knowledge quiz had passing knowledge of refined mouse handling with an average quiz score of 75%. Surely 75% reflects more than a 'passing' knowledge? Please define what you mean by passing – number of correct responses?
Line 508: should this be 21000 or 2100?
Line 573 reads awkwardly: "the advantages refined handling provides both the mice and people". I suggest changing this to 'the advantages refined handling provides both to the mice and to the people handling them'
Line 574: Change "this is…" to 'this was…'
Figure Legends. Figure 6 refers to left and right bars, whereas figures 7 and 8 refer to light and dark bars. I don't see them as either dark or light, they are just different colours. Be consistent.
Lines 699-701: I found this a little confusing. Why would the cost or tunnels be included as an enabling factor when the respondent mentioned personnel, autoclave capacity and ended with 'unfortunately money'. Surely this comment is related to barriers rather than enablers.
Line 75: 'preferred' would be better than 'personal methods'.
Line 756: you don't need both 'overall' and 'generally' in the same statement.
Line 785-787: change 'institutional' to 'institution'. Overall, this sentence could be written better as the current meaning is unclear.
Line 792: 'in this survey' is an obsolete remark.
Line 786: 'despite that refined…' should be 'despite the fact that..'
Line 800: they're – they are
Line 869-870: Avoid references to this being a 'study' when it's a survey.
Line 873-4: what does 'skewed to individuals' mean.
Line 876: 'in this survey' is also an obsolete remark.
Line 893: 'requiring' should be 'requires'
