# Supplementary material for: Using refined methods to pick up mice: A survey benchmarking prevalence & beliefs about tunnel and cup handling
Source: PLoS One. 2023 Sep 7;18(9):e0288010. doi: 10.1371/journal.pone.0288010 (PMC10484434; doi:10.1371/journal.pone.0288010)
Supplement: Attachment — Submitted filename: Response To Reviewers.docx [file pone.0288010.s004.docx]

June 2, 2023

Dear Dr. Cord Brundage,

Thank you for inviting us to submit a revised version of our manuscript. We appreciate the detailed and helpful feedback that both reviewers provided. We believe their contributions have strengthened our manuscript. We have completed all revisions and address each comment individually below. The reviewer's comments are bolded and our responses are non-bolded. Quotes from the manuscript are italicized.

We look forward to receiving your response and decision. Thank you for inviting our revision.

Sincerely,

Megan LaFollette, MS, PhD and Co-Authors

**Journal requirements:**

**When submitting your revision, we need you to address these additional requirements.**

**1. Please ensure that your manuscript meets PLOS ONE's style requirements, including those for file naming. The PLOS ONE style templates can be found at**

[**https://journals.plos.org/plosone/s/file?id=wjVg/PLOSOne_formatting_sample_main_body.pdf**](https://journals.plos.org/plosone/s/file?id=wjVg/PLOSOne_formatting_sample_main_body.pdf) **and**

[**https://journals.plos.org/plosone/s/file?id=ba62/PLOSOne_formatting_sample_title_authors_affiliations.pdf**](https://journals.plos.org/plosone/s/file?id=ba62/PLOSOne_formatting_sample_title_authors_affiliations.pdf)

We believe we have fixed a few style requirements and file naming requirements

**2. Please provide additional details regarding participant consent. In the ethics statement in the Methods and online submission information, please ensure that you have specified what type you obtained (for instance, written or verbal, and if verbal, how it was documented and witnessed). If your study included minors, state whether you obtained consent from parents or guardians. If the need for consent was waived by the ethics committee, please include this information.**

We have added to the ethics statement, *“All procedures, informational sheet, and online waived signed consent protocols were reviewed and determined to be exempt from ongoing review by University of Michigan’s Human Research Protection Program Institutional Review Board (IRB), protocol #HUM00195571.*

We have added this information to the methods: “*Participants were provided with an online information sheet to read prior to the study that assured them responses would be kept confidential and they were asked to document waived signed consent via a yes or no question.”*

**3. We note that you have stated that you will provide repository information for your data at acceptance. Should your manuscript be accepted for publication, we will hold it until you provide the relevant accession numbers or DOIs necessary to access your data. If you wish to make changes to your Data Availability statement, please describe these changes in your cover letter and we will update your Data Availability statement to reflect the information you provide.**

This is still correct.

**4. Please review your reference list to ensure that it is complete and correct. If you have cited papers that have been retracted, please include the rationale for doing so in the manuscript text, or remove these references and replace them with relevant current references. Any changes to the reference list should be mentioned in the rebuttal letter that accompanies your revised manuscript. If you need to cite a retracted article, indicate the article’s retracted status in the References list and also include a citation and full reference for the retraction notice.**

There are no changes to our reference list.

**Reviewer: 1**

1. **This submission represents a survey of current mouse handling practices, primarily in the USA, and concludes that much additional education is needed to promote wider use of refined handling. While I agree with the methods and conclusions, my only major grip is that statement to that effects are repetitive, especially in the discussion and what is said in the final conclusion. I feel the manuscript could be significantly shorter and the wording more concise. Other issues to address are listed below, but these are generally only minor issues.**

Thank you to reviewer 1 for their helpful and constructive comments. We have made the entire paper more concise, especially the discussion and conclusion.

2. **The figures in Table 1 under role add to 98% not 100%. Are these rounded, if not, what happened to the missing 2%?**

These are rounded to the nearest full percent (and all but one are less than 0.5%) so that’s what happened to the missing 2%. We’ve added this note in the table header.

3. **In the individual practices section, it is stated that 215 participants reported on the methods they personally use to pick up mice. Why then, does Figure 5 only report data from 194 participants? Was this just because not all answered those questions?**

This discrepancy is because only 194 participants answered the knowledge quiz questions that are displayed in the results of Figure 5. This is mentioned in the text, but we have added clarification to the Figure 5 caption. *“This graph displays the percentage of participants who answered the knowledge quiz (n = 194)...”*

4. **Lines 423-424 say participants that answered the knowledge quiz had passing knowledge of refined mouse handling with an average quiz score of 75%. Surely 75% reflects more than a ‘passing’ knowledge? Please define what you mean by passing – number of correct responses?**

Thank you for this comment. We have changed the wording of this sentence from “passing” to “adequate” to reduce confusion about what is meant. *“The 194 participants that answered the knowledge quiz had adequate knowledge of refined mouse handling with an average quiz score of 75%”*

5. **Line 508: should this be 21000 or 2100?**

We reported this directly as stated in the participant response, but this has been clarified to *“2100”* assuming the respondent did not mean to add a comma.

6. **Line 573 reads awkwardly: “the advantages refined handling provides both the mice and people”. I suggest changing this to ‘the advantages refined handling provides both to the mice and to the people handling them’**

Thank you for this suggested revision. This has been modified as suggested to state *“Participants also mention the advantages refined handling provides both to the mice and to the people handling them.”*

7. **Line 574: Change “this is…” to ‘this was…’**

This has been modified.

8. **Figure Legends. Figure 6 refers to left and right bars, whereas figures 7 and 8 refer to light and dark bars. I don’t see them as either dark or light, they are just different colours. Be consistent.**

Thank you for this catch. We have modified the Figure 7 and Figure 8 captions to both state, *“Left bars indicate individual-level responses (n=X), right bars indicate institutional-level responses (n=X)”*

9. **Lines 699-701: I found this a little confusing. Why would the cost or tunnels be included as an enabling factor when the respondent mentioned personnel, autoclave capacity and ended with ‘unfortunately money’. Surely this comment is related to barriers rather than enablers.**

Thank you for this comment. We agree and have removed this quote to avoid confusion.

10. **Line 75: ‘preferred’ would be better than ‘personal methods’.**

Thank you for the suggested revision, this has been modified.

11. **Line 756: you don’t need both ‘overall’ and ‘generally’ in the same statement.**

Thank you to the reviewer for catching this. The word *“generally”* at the end of this sentence has been removed.

12. **Line 785-787: change ‘institutional’ to ‘institution’. Overall, this sentence could be written better as the current meaning is unclear.**

Thank you for this comment, we agree that the meaning of this sentence, as it is currently worded, may be unclear. We were hoping to convey the contrast between the individual responses on this topic, mentioned in the previous sentence, to the institutional responses. For individuals, those who reported feeling confident in implanting refined handling did not report higher levels of planned future use of refined handling. Conversely, this relationship was reported when participants were asked about their institution’s practice.

We have changed ‘institutional’ to ‘institution’ and have reworded this sentence to say, *“Conversely, participants that felt confident their institution could implement refined handling reported higher future institutional practices, and also reported high current institutional practices”*

13. **Line 792: ‘in this survey’ is an obsolete remark. Line 786: ‘despite that refined…’ should be ‘despite the fact that..’ Line 800: they’re – they are**

Thank you for making these suggestions. We have made these three wording changes to the mentioned sentences.

14. **Line 869-870: Avoid references to this being a ‘study’ when it’s a survey.**

Thank you for this comment. We have reviewed the entire discussion section and changed our wording from ‘study’ to ‘survey’.

15. **Line 873-4: what does ‘skewed to individuals’ mean.**

Thank you for this comment. We were aiming to convey that our survey may have specifically targeted individuals who feel passionately about refined handling, which may be a study limitation. We have modified the wording of this sentence for clarity to state, *“Additionally, our respondent pool may have been biased towards individuals who felt particularly passionate either for or against refined handling.”*

16. **Line 876: ‘in this survey’ is also an obsolete remark.**

Thank you for catching this and ‘in this survey’ has been removed from the end of this sentence.

17. **Line 893: ‘requiring’ should be ‘requires’**

This has been corrected.

**Reviewer 2:**

1. **Overall, the study is well designed, analysed and findings reported quite clearly. There are a few issues that need clarification prior to publication. My review focuses on some general issues before listing some specific issues of phrasing. Points are numbered for convenience of response.**

Thanks to the reviewer for these comments.

**There is some confusion in the section of Results benchmarking institutional practices (p18-19) and individual practices (p21) – these are key data to understand, so important to report this as clearly as possible. The survey asks separate questions about what methods are approved for use at an Institutional level, what methods are used by individuals, and what percentage of mice are picked up by non-aversive methods ‘as default’ by Institutions and by individual respondents (it would help to define what ‘as default means here).**

Thank you for catching this. We have added a definition for ‘default method’ after the first time this term is mentioned in our manuscript.

**Fig 3 is labelled current handling methods used by individuals and institutions. There are two different Fig legend titles in the manuscript I am reviewing, but the legend indicates “Laboratory animal personal were asked to report the methods used to handle mice on behalf of their institution”. The text however cites Fig 3 as showing the methods approved at an institutional level for picking up mice from their cages, not what is used (line 350), and Fig 3 as showing the methods personally used by respondents (line 394). While it is ok to show different measures of methods allowed and used in the same figure, this needs to be clearly communicated. It took me a lot of digging to work it out.**

Thank you for this comment. In figure 3, the institutional (orange) bars represent handling methods approved at the individual’s institution, and the individual (blue) bars represent handling methods used by the individual. This has been made more clear in the Figure 3 caption by stating,

*“****Fig 3.*** ***Handling methods for individuals and institutions.*** *Laboratory animal personnel were asked to report the approved methods used to handle mice on behalf of their institution (n = 103) and the methods used by themselves personally (n = 215).”*

2. **The study particularly focuses on the percentage of institutions and individuals that use refined methods exclusively and seems to make the assumption that this is the desirable situation and should have high prevalence. However, is there background evidence to support the proposal that refined methods are appropriate in all situations? For example, respondents report that tunnel and cup methods may not be suitable for safety reasons in some situations, or may not be appropriate for use in isolators. I am not familiar with published evidence that refutes this or shows how the methods can be used effectively in isolators and I think exclusive use needs a little more careful consideration, particularly in Discussion (lines 737-747). This is not to argue that the prevalence of current use can be explained by such specific situations, but queries whether exclusive use is the most relevant measure to emphasise rather than the proportion of animals handled.**

From our review of the literature and discussions with real world implementation, we believe it is desirable for refined methods to be used as the default method, with tail handling/forceps/scruffing only approved in very unique circumstances. Even in isolators, there’s been the discussion of using two tunnels or a close-ended tunnel to move mice without touching them (and some UK laboratories already use handling tunnels in isolators).

From this survey, it’s clear this is not the case. To make this clear we’ve added the sentence to the section referenced of, *“Additionally, our participants estimated only a median of 10% of mice at their institution were handled with refined mouse handling.” We’ve also changed “exclusive” to “default” use in the next paragraph.*

3. **It is also important to report data that allow valid comparisons. In the text, the % of institutions and % of individuals that use refined methods exclusively are given. Then it states “Conversely, nearly all institutions allowed tail handling” (line 353) or “Conversely, nearly all individuals used tail handling” (line 396). This is not a converse (opposite) comparison. How many institutions / individuals used tail handling exclusively by comparison? Then you can compare what proportion used refined methods versus used tail handling, and the estimated proportion of mice handled by tail or refined methods. This is important to understand what proportion of institutions are failing to allow the refined methods as an approved method, or individuals that are completely avoiding their use, rather than using a mixture of methods.**

We thank the reviewer for this comment and have added sections to point out where only tail handling was allowed/used and added the percentages of each. This has strengthened the manuscript. See below:

*“Only 5% of institutions allowed only refined methods for picking up mice (e.g., tunnel handling and/or cupping should be used, tail handling is prohibited). Conversely, 18% of institutions allowed only tail handling and/or forceps handling (e.g., tunnel handling and tail handling were not allowed). In terms of allowed techniques, 92% of institutions allowed tail handling, 75% allowed cupping, 68% allowed tunnel handling, 48% allowed tunnel handling, 47% allowed forceps, and 5% allowed scruffing.”*

*“Only 10% of individuals used only refined methods to pick up mice. Conversely, 37% of individuals used only tail handling, forceps, and/or scruffing (e.g., they never used tunnel or cupping). In terms of the techniques used at least some of the time, 89% used tail handling, 55% used cupping, 41% used tunnel handling, 16% used forceps, and 5% scruffed.”*

4. **Reporting the actual data will help avoid vague statements. For example in line 718-719 of the Discussion “Our results indicated that despite our participants indicating they had high familiarity with refined handling that it is relatively infrequently used.” But what does relatively infrequently used mean? The dictionary definition of infrequent is ‘not happening very often or rare’, but respondents estimated they picked up 18% of mice with refined methods, which doesn’t seem rare, and may be doing this regularly for some mice. A more meaningful statement could be, for example, “Despite 70% of participants reporting moderate or strong familiarity with refined handling, they report that they handled only a median of 18% of mice with these methods.” This is unambiguous. Similarly, see Conclusions line 887.**

We appreciate these comments, however we used this terminology in the discussion to avoid excess repeating of the results, summarizing, and a concise discussion (especially at reviewer 1’s encouragement to make this section more concise). Still, we have made a few of the suggested modifications using actual data to make it more explicit through the discussion.

5. **Some of the comments under the qualitative analysis suggest that respondents did not always understand the handling methods to pick up mice that this study specifically focused on, which should be discussed briefly. For example, the comment “usually looking at mice for medical examination and/or treatment and need to restrain animals so they will not bite me during examination and treatment” suggests that the respondent was not aware they could restrain the animal once picked up (line 491-3). The comments that “Animal care techs are worried about injuring mice if they use a cup” and “[it is] difficult to disinfect tunnels or cups/scoops thoroughly” (line 591-20) suggest that some respondents used physical cups for handling rather than cupped hands although this was the focus of the survey.**

We thank the reviewer for this comment. We agree with the interpretation that even though participants believed they were familiar with the technique, they had a number of misconceptions about it. The misconception that mice cannot be restrained after using refined handling is addressed in the Discussion – Misconceptions, Time, & Materials required for Handling.

6. **In the generalised linear regression analyses to establish which explanatory variables are associated with implementation of refined handling, it is not clear why specific comparisons are made between particular categories when explanatory variables had multiple categories and how these comparisons were chosen for inclusion (Tables 3 and 4). For example, the influence of location was compared between USA vs all other locations pooled, and the effect of Institution type was compared between Academic vs all others pooled. But the effect of role was compared specifically between Manager vs Veterinarian (not between the most frequent category Veterinarian vs all other categories pooled, while there were as many caretaker respondents as managers). Please provide further explanation and justification in the Data Analysis section to show whether comparisons were chosen to address specific questions of interest, were based simply on the distribution of data (most frequent category vs the rest), or were selected post hoc because there looked to be a difference between specific categories.**

For Location and Institution, the sample size was too small and unbalanced to allow highly granular comparisons for this multidimensional data set. After preliminary screening of category sample sizes, a priori collapsing of variable categories was necessary to allow model fit and obtain interpretable results. For Location, dichotomising as USA vs Other was the most sensible choice: there were 205 responses for USA vs Canada (23), Europe (24), and Other (9). For Institution, the categories were Academic (158), Industry (65), Other (government, CRO; 45).

For Profession, there were six categories: Veterinarian, Researcher, Manager, Technical, Caretaker, Other. Initially all categories were entered into the model: the choice of which comparisons to drop from the final model was performed by the LASSO method. LASSO is a regression analysis method that performs both variable selection and regularization to enhance prediction accuracy and interpretability of the resulting model. the coefficients are estimated by penalised logistic regression; the log-likelihood function is maximized with a penalty function on coefficient size. The penalty function is the Least Absolute Shrinkage Selection Operator (LASSO). LASSO gets more degrees of freedom by trying to select only one or a few items among a group of correlated items and removing insignificant variables. For Profession, there were six categories: Veterinarian, Researcher, Manager, Technical, Caretaker, Other. Veterinarian was the reference category; because these categories were relatively well-balanced, each profession was compared to Veterinarian.

[ Example:

| **Term** | **Estimate** | **Std Error** | **Wald ChiSquare** | **P** | **Lower 95%** | **Upper 95%** |
| --- | --- | --- | --- | --- | --- | --- |
| Intercept | -0.29 | 0.17 | 3.017 | 0.08 | -0.63 | 0.04 |
| LOC_USA | -0.08 | 0.07 | 1.35 | 0.25 | -0.21 | 0.05 |
| PROF_ROLE[MAN-VET] | 0.02 | 0.057 | 0.11 | 0.74 | -0.09 | 0.13 |
| PROF_ROLE[MAN-VET] | 0 | 0 | 0 | 1 | 0 | 0 |
| PROF_ROLE[OTHER-VET] | 0 | 0 | 0 | 1 | 0 | 0 |
| PROF_ROLE[RES-VET] | 0 | 0 | 0 | 1 | 0 | 0 |
| PROF_ROLE[TECH-VET] | 0 | 0 | 0 | 1 | 0 | 0 |
| Years_WorkMice | 0 | 0 | 0 | 1 | 0 | 0 |
| Institution[ACADEM-OTHER] | 0 | 0 | 0 | 1 | 0 | 0 |

We have added an explanation of the variable selection method in the methods section as follows *“To enhance prediction accuracy and interpretability of the models, variables were selected using the Least Absolute Shrinkage Selection Operator (LASSO). This is an extension of ordinary least squares regression where regression coefficients are estimated by adding a penalty function (the LASSO) to the residual sum of squares. The penalty function results in those regression coefficients that are least associated with the response variable being decreased to zero, which effectively removes them from the model.” …*

*“In the regression, demographic categories were collapsed into larger categories prior to analysis to assist with unbalanced and small sample sizes. Location was dichotomized into USA and Other. Institution was collapsed into the categories were Academia, Industry, and Other. Location was collapsed into categories (Veterinarian, Researcher, Manager, Technician, Caretaker, Other) that were entered into the model. The choice of which comparisons to drop from the final model was performed by the LASSO method described above with Veterinarian as the reference category.”*

7. **Explanation of figures should be in the figure legend without an additional title above.**

We prefer to have the figure explanation in both the figure legend and the actual figure. This is to assist people in quick interpretation of a figure without having to read the legend (and as some people may skip reading the legend altogether). However, to allow better use of screen readers we have moved a duplicate of the interpretation to the figure legend as necessary.

8. **There was a problem with formatting of Table 3 in my version, and Tables 1 & 2 are unlikely to meet the formatting requirements of the journal (no boxes). The contrast of colors in Fig 5 was poor and would be difficult for anyone with impairment to see.**

We thank the reviewer for catching this. We have re-formatted Tables 1-3 to align with the formatting requirements for PLOS One. We have also changed the colours in Figure 5 to be accessible for individuals with visual impairments.

9. **The paper is quite long and unnecessarily wordy in places. The paper will have maximum impact if people can quickly get to the key points and findings. I would encourage the authors to cut out unnecessary words and phrases, and check punctuation carefully.**

We have reviewed the entire manuscript for conciseness and cut a number of words and phrases. We also believe our use of headers will help individuals quickly navigate to the sections of interest. **Specific comments**

10. **Abstract line 27-8: ‘yet widespread implementation seems to be low’. Hard to see how widespread implementation can be high or low. Do you mean ‘yet implementation does not seem to be widespread’?**

The suggested change has been made.

11. **Abstract line 28-9: There are other handling methods or combinations of methods that have also been shown to be refinements (for pick up or restraint). Suggest you amend to ‘Refined handling includes ….’**

The suggested change has been made.

12. **Abstract line 31: A hypothesis is normally a proposed explanation for a phenomenon that can be tested scientifically. Instead, these appear to be predictions. What is meant by ‘low’ (need to know that or you cannot assess the prediction). Do you mean compared to traditional tail handling?**

Both of these comments have been addressed and modified in the Abstract lines 31-33.

13. **Abstract line 41: As above ‘low levels’ are not defined. Would be much more meaningful to cite some specific data as in general comment 4 above. Surely it is more useful to understand how many respondents / institutions are using refined methods and on what proportion of animals compared to traditional methods than whether or not they are used exclusively? (particularly if exclusive use is not proven to be a good thing).**

We’ve addressed our opinions on exclusive use above and cited both “*with only 10% of participants using it exclusively and a median estimate of only 10% of institutional mice being handled with refined methods.*” We used this wording to try to be concise in the abstract due to word count limitations.

14. **Intro line 71: Should point out here or elsewhere that other similar methods can also provide a refinement for mice such as picking up mice on a cage ladder (reference 27) or cupping on the hands with massage (reference 13).**

We thank the reviewer for making this helpful comment. We have made this more explicit by adding a line to the methods that states, *“Of note, there are additional refinements to tail handling that were not covered in this survey, including picking up mice on a cage ladder (27) and cupping on the hands with massage (13).”*

15. **Intro line 85: You should cite the study that originated the methods here (reference 8).**

This was an oversight, thank you to the reviewer for catching this. This has been corrected.

16. **Intro line 110-113: The use of ‘aims’ and ‘objective’ seem to be the wrong way round here. Aim refers to the broad intent, objectives are the steps taken to achieve that.**

This has been corrected.
